# Utility of endometrial sampling prior to risk-reducing hysterectomy in a patient with Lynch syndrome

**DOI:** 10.3332/ecancer.2016.613

**Published:** 2016-01-18

**Authors:** Melissa K Frey, Gizelka David-West, Khushbakhat R Mittal, Franco M Muggia, Bhavana Pothuri

**Affiliations:** 1 Department of Obstetrics and Gynecology, Division of Gynecologic Oncology, New York University Langone Medical Center, New York, NY 10016, USA; 2 Department of Pathology, New York University Langone Medical Center, New York, NY 10016, USA; 3 Department of Medicine, New York University Langone Medical Center, New York, NY 10016, USA

**Keywords:** colorectal neoplasms, hereditary nonpolyposis, endometrial neoplasms, hysterectomy, risk reduction surgery, uterine neoplasms

## Abstract

Occult endometrial cancer is occasionally discovered in women with Lynch syndrome undergoing risk-reducing hysterectomy. The case presented here demonstrates that preoperative endometrial sampling can help detect these occult cancers; however, there are currently no recommendations for this preoperative intervention. A 50-year-old woman with Lynch syndrome underwent endometrial sampling prior to planned risk-reducing hysterectomy and bilateral salpingo-oophorectomy. The endometrial biopsy demonstrated a serous endometrial cancer. The patient was counselled regarding the diagnosis and revised operative plan, which now included staging, prior to surgery. Although the prevalence of occult endometrial cancer at the time of risk-reducing surgery in women with Lynch syndrome remains unknown, preoperative endometrial sampling may allow for improved patient counselling and surgical planning in this population, and can help avoid a subsequent surgery for staging.

## Introduction

Women with Lynch syndrome have an autosomal dominant mutation in one of the DNA mismatch repair genes resulting in an increased lifetime risk for colorectal and endometrial cancer in addition to cancers of the ovary, stomach, hepatobiliary tract, pancreas, small bowel, urinary tract, and central nervous system. The current National Comprehensive Cancer Network (NCCN) guidelines recommend that individuals with Lynch syndrome undergo colorectal cancer screening with colonoscopy [1]. There is less data to support gynaecologic screening. However, because of high lifetime risk of endometrial cancer in women with Lynch syndrome the NCCN and other consensus guidelines recommend annual or biennial endometrial sampling beginning at age 30–35 years and risk-reducing hysterectomy and bilateral salpingo-oophorectomy in women who have completed childbearing [1, 2]. The role of endometrial sampling preceding risk-reducing surgery is yet to be addressed. We present a case of an asymptomatic patient found on preoperative endometrial sampling to have serous endometrial cancer, raising the question of whether preoperative endometrial sampling should become the standard of care for Lynch patients undergoing risk-reducing hysterectomy.

## Case presentation

A 50-year-old, gravida zero, Ashkenazi Jewish woman underwent screening for Lynch syndrome. Her paternal grandfather was diagnosed with colon cancer at age 80, paternal uncle diagnosed with colon cancer at age 50, and two paternal second cousins diagnosed with premenopausal endometrial cancer. Both the patient and her father were found to have the Lynch syndrome mutation MSH6 3959del4, which results in premature truncation of the MSH6 protein at amino acid position 1325 [3]. Following this screening result, the patient was immediately referred for risk-reducing gynaecologic surgery.

**Figure 1.**
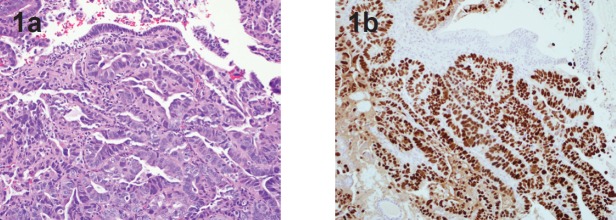
1a. Glandular variant of uterine serous carcinoma. Notice the high grade nuclei, H & E 20x. 1b. Immunostain for p53 highlights all tumor nuclei. Benign epithelium and stroma are not stained. p53 immunostain 20x.

The patient had no personal history of cancer or gynaecologic pathology and reported regular menses with a 20-year history of oral contraceptive use. The patient underwent a pelvic ultrasound which demonstrated an 8 mm endometrial echo with heterogeneity, likely representing small benign endometrial polyps. She was referred to a gynaecologic oncologist who counselled her regarding the option for risk-reducing surgery, which would include total hysterectomy and bilateral salpingo-oophorectomy. The gynaecologic oncologist performed an endometrial biopsy in the office after explaining to the patient that despite normal menses and a lack of gynaecologic symptoms, a preoperative diagnosis of endometrial cancer would affect the surgical plan and therefore a preoperative endometrial biopsy could provide valuable information. The endometrial biopsy demonstrated endometrial serous adenocarcinoma (Figure 1). The patient underwent a preoperative CT scan which showed no evidence of metastatic disease. She was counselled that based on the preoperative pathologic diagnosis, the recommended surgery now included pelvic and para-aortic lymph node dissection, omentectomy, and peritoneal biopsies.

The patient underwent an uncomplicated laparoscopic total hysterectomy, bilateral salpingo-oophorectomy, pelvic and para-aortic lymph node dissection, omentectomy, and peritoneal biopsies. The final pathology demonstrated a stage IA uterine papillary serous cancer with no myometrial invasion. The patient received three cycles of chemotherapy with carboplatin and paclitaxel with no evidence of disease at the conclusion of treatment. She will continue screening for other Lynch-associated cancers.

## Discussion

Women with Lynch syndrome undergoing risk-reducing hysterectomy are at risk for having an occult endometrial cancer, however the magnitude of risk remains unknown. The estimated cumulative lifetime risk for endometrial cancer in Lynch syndrome varies by report and mutation, ranging from 21–71%. The MSH6 3959del4 mutation (as in the current case) is associated with the greatest risk of endometrial cancer, affecting 71% of women by age 70 [3]. Prior case reports have described occult endometrial cancer discovered in patients with Lynch syndrome undergoing risk-reducing surgery [4, 5]. Lachiewicz *et al* [6] recently published the first study to explore the prevalence of occult gynaecologic malignancy at the time of risk-reducing surgery in patients with Lynch syndrome. Four of 24 (16.7%) patients in this study were noted to have cancer on final pathology, three with endometrial cancers (12.5%), and one with ovarian cancer (4.2%).

Currently, there are no recommendations in place for endometrial sampling prior to risk reducing surgery and, in fact, gynaecologic cancer surveillance in Lynch syndrome patients remains an area of debate. Two studies have reported a lack of efficacy of screening transvaginal ultrasounds in women with Lynch syndrome [7, 8]. More recently, however, Lécuru *et al* [9] found that ultrasonography alone was effective for diagnosing endometrial cancer with 100% sensitivity and 100% negative predictive value. In the case presented here, the ultrasound report findings showed what appeared to be benign endometrial polyps. The results of studies evaluating screening endometrial sampling have been similarly conflicting [8, 10]. Women with Lynch syndrome have a high lifetime risk of endometrial cancer and many will develop the disease prior to menopause when irregular bleeding may not be recognised as an early symptom [11]. As a result, despite conflicting results of screening efficacy, the NCCN, American Congress of Obstetricians and Gynaecologists, and other consensus groups recommend endometrial cancer surveillance as an option in this high-risk population [1, 2].

The reported cases of occult endometrial cancer discovered at the time of risk-reducing hysterectomy pose the question of whether endometrial sampling should be a routine component of the preoperative evaluation. Our case is another example of an occult endometrial cancer but additionally it highlights the clinical benefit of obtaining information on a cancer diagnosis prior to surgery. The patient described in this case was diagnosed with a serous endometrial cancer prior to surgical intervention. Patients with Lynch syndrome can have either type I (endometrioid) or type II (high-risk histologic subtypes) endometrial cancers. Broaddus *et al* [12] performed a pathologic evaluation of 50 Lynch-associated endometrial cancers and found that 14% were type II. Furthermore, serous endometrial cancers in this population present at an earlier age than in the general population (46 years versus 65–68 years), as it occurred with our patient [12, 13]. Risk-reducing hysterectomy and bilateral salpingo-oophorectomy is an incomplete surgery for serous endometrial cancers and therefore patients with occult disease diagnosed only on final surgical pathology will require a second surgery. Because of the preoperative diagnosis, our patient was able to receive thorough preoperative counselling about the high-risk pathology and surgical plan, and she underwent complete surgical staging in a single procedure, which likely would not have been possible without the preoperative diagnosis of endometrial cancer. This patient was already being followed by a gynaecologic oncologist but many patients undergoing risk-reducing surgery will have their surgery performed by general gynaecologic surgeons. For these patients, a positive preoperative biopsy will allow for early referral to subspecialty care.

## Conclusion

Further prospective studies are required to determine the true prevalence of occult endometrial cancers at the time of risk-reducing surgery. However, in the meantime, surgeons may want to consider preoperative endometrial sampling in women with Lynch syndrome undergoing risk-reducing surgery, especially if in a small cohort the incidence was as high as 12.5%. As in this case, the diagnosis of cancer enabled a pre-operative discussion with the patient, and this in turn helped to avoid a second surgery for staging.

## Conflicts of interest

There are no conflicts of interest to report.
